# The R.M.O. and the County Court

**Published:** 1908-01-04

**Authors:** 


### Resident Medical Officers' Department.

[The Editor accepts no responsibility for any opinions expressed in these columns, which are freely open to all resident medical officers. Discussion and contributions are invited, and, if the latter are accepted, they will be paid for.]

### THE R.M.O. AND THE COUNTY COURT.

### I. THINGS TO KEEP IN MIND.

As a rule it is when the newly qualified practitioner undertakes the duties of a resident medical officer that he is first brought into close and frequent contact with the mysteries of the law. The Coroner's Court, the Police Court, the County Court, and even more august tribunals still, are certain to require his presence before he has been many weeks in office; and there are numerous "wrinkles" to be learnt about each of them which only experience or attention to the experience of others can teach. The ordinary text-books of medical jurisprudence are concerned with the exposition of various laws and decisions bearing on different forms of crime, and do not deal with such matters as civil causes and the position of the medical witness with regard to them.

It is as witness in compensation claims for injuries due to accidents that the resident medical officer, and indeed for that matter the practitioner too, will most often attend at a County Court. He may appear for the plaintiff in support of such a claim, or for the defence in rebutting it. In either case he stands in a position which in one respect is a dual one: that is, up to a certain point he is a common witness, or witness of facts; while beyond that, and much more important, he is an expert witness who by his professional skill interprets for the Court the results of his special observations.

Now this distinction is a material one. An expert witness need not accept a subpœna from a litigant unless he so chooses; in other words, unless he receives what he considers sufficient remuneration for the time and trouble involved. A common witness, on the other hand, if with a subpœna he is tendered the recognised "conduct money," is obliged to attend. It is true that he is not liable by non-attendance to a charge of contempt of Court, as he would be in a criminal case; but he is liable to a civil action for such damages as the litigant may have suffered by his failure to give evidence.

Consequently the R.M.O. who admits a case of accident to his wards, or even treats it as an out-patient casualty, is to a limited extent a common witness. Of course, when he asserts his position as an expert, that position is at once conceded by the solicitors in charge of the suit; but they are always anxious to procure his evidence on the lowest possible terms, and they will often try to "bluff" an inexperienced or unwary house surgeon into giving expert evidence for the statutory common witness fee. Supposing the unlikely event were to happen of a solicitor persisting in this attitude, the witness must explain the circumstances to the judge before being sworn, and refuse to answer any questions except those of simple fact. Thus the date of admission of the patient and the length of his sojourn in the hospital are obviously common evidence. The estimate of the time during which he has been totally or partially disabled and the prognosis of any still existing result of an accident are as obviously expert evidence. But other points are not so clear: whether questions as to the occurrence of a fracture, the methods of treatment, the amount of suffering endured by the patient, and so on, would be permitted by the judge is difficult to say, for the line is not an easy one to draw. However, the situation is not likely to occur.

The usual but not by any means invariable preliminary to the issue of a subpœna is a request for a report on the medical aspects of the particular case. This, of course, is a signed statement the details of which one may be called upon to repeat in the witness-box. It should therefore be furnished only after due deliberation, and nothing debateable should be included in it. An additional reason for care is the possibility of cross-examination upon it, and the report should in consequence be drawn up in the simplest and clearest possible form, so as to leave no loophole for misinterpretation. Many cases are compromised without litigation at all, simply upon the house surgeon's report, and therefore a fee should always be charged, and the transaction should be on a cash basis: solicitors sometimes ask for a report gratis, on the ground that the surgeon will get a fee as a witness later. It is advisable to mention also not only the fee for the report, but also that required for any subsequent attendances in Court. A duplicate of the report should be kept in every case.

The fee for this report is, of course, at the discretion of the medical officer. At some hospitals it is fixed by the rules at a guinea, and at a few others there is provision that all such fees are to be handed over to the funds of the charity. Speaking generally, a guinea is a fair minimum for County Court cases, and less should never be accepted. Solicitors of a certain class will often resist this demand, and appeal to the charity of the house officer on the plea that their client is a poor man who cannot afford such a sum, or any fee at all. This is especially the case when the solicitor has taken up the case for the plaintiff on the understanding that the latter pays no law costs in any case, while the former takes half the profits if a verdict is obtained. It may be remarked that the system is improper, if not actually illegal, but it is frequently practised. Its exponents are always ready to sweat the young house surgeon. Such solicitors sometimes offer to pay for the report if their client proves successful: the offer is made by a junior clerk whom his employers can disown if necessary, for any such arrangement will, if discovered, bring both lawyer and doctor deservedly under the censure of the Court.

A report may be asked for by both sides, and there is no reason why both should not be supplied, and two fees accepted. The receipt of a fee from one party to an action does not, or should not, constitute the medical witness a partizan: his proper position is that of an expert who has special knowledge of the case, and puts it at the service alike of the Court and of the litigants.

